# A diagnostic dilemma: Pedunculated mesenteric lipodystrophy mimicking Meckel’s diverticulum. A case report and literature review

**DOI:** 10.1016/j.ijscr.2020.05.083

**Published:** 2020-06-09

**Authors:** Alessandra Mirabile, Marco Moschetta, Nicola Lucarelli, Michele Telegrafo, Arnaldo Scardapane, Amato Antonio Stabile Ianora

**Affiliations:** a DIM, Interdisciplinary Department of Medicine, Section of Diagnostic Imaging, Aldo Moro University of Bari Medical School, Piazza Giulio Cesare 11, 70124 Bari, Italy; b DETO, Department of Emergency and Organ Transplantation, Aldo Moro University of Bari Medical School, Piazza Giulio Cesare 11, 70124 Bari, Italy

**Keywords:** Mesenteric lipodystrophy, Mesenteric panniculitis, Sclerosing mesenteritis, Meckel’s diverticulum, Computed tomography, CT

## Abstract

**Introduction:**

Mesenteric lipodystrophy is a rare fibroinflammatory disease of unknown origin with clinical and radiological non specific findings.

**Presentation of the case:**

The case of a 42-years-old man affected by a pedunculated mesenteric lipodystrophy mimicking Meckel’s diverticulum is reported. Clinical, imaging and histological findings are discussed.

**Discussion:**

Mesenteric lipodystrophy affects the mesenteric fat of the abdomen with a typical diffuse thickening of the mesentery, nodular thickening of the mesenteric root and presence of mass-like lesions. Ultrasound (US) and Multiphasic Computed Tomography (CT) represent the main imaging tools used for diagnosis. Clinical and imaging findings can mimick other pathological conditions affecting the mesenteric fat tissue. Contrast enhanced CT is the most accurate imaging technique for diagnosing mesenteric lipodystrophy due to the high panoramicity and accuracy with multiplanar imaging. Multiphasic technique helps to characterize the lesion and to recognize vascular anatomy. Oral administration of iodinated contrast medium may help to assess the relationship with bowel loops. All these diagnostic elements are crucial for the surgical timing and approach.

**Conclusions:**

Due to the heterogeneous mesenteric involvement, the nonspecific CT findings and the high number of diseases for differential diagnosis, the detection of mesenteric lipodystrophy is challenging and requires early clinical suspicion. An histological examination is always necessary.

## Highlights

• Mesenteric lipodystrophy is a rare fibroinflammatory disease of unknown origin.
• The detection of mesenteric lipodystrophy is challenging and requires early clinical suspicion.
• Clinical and imaging findings can mimick other pathological conditions affecting the mesenteric fat tissue.
• Contrast enhanced CT is the most accurate imaging technique for diagnosing mesenteric lipodystrophy.

## 1 Introduction

Mesenteric lipodystrophy is a rare fibroinflammatory disease of unknown origin with just over 200 cases described in the medical literature [1].

Since its first description in 1924, several terms have been used to describe this condition, including mesenteric panniculitis, sclerosing mesenteritis, retractile mesenteritis, mesenteric lipogranuloma. Nowadays most authors accept that these different entities represent a spectrum of the same disease.

The etiology is unclear; however, previous abdominal trauma, surgery, mesenteric ischemia or infection have been proposed as potential causes [2].

Clinical onset is subtle, with nausea, abdominal tenderness, fever, asthenia. Computed tomography (CT) represents the most accurat examination to achieve diagnosis being crucial also to define any surgical approach. However, CT findings are often nonspecific and need to be correlated to the clinical information and often requires biopsy and histological examination for a definitive diagnosis [[1], [2], [3]]. The work has been reported in line with the SCARE criteria [4].

## 2 Presentation of the case

A 42 years-old Caucasian man came to our emergency department with abdominal tenderness in the lower right abdominal quadrant and elevation of blood index of inflammation.

**Fig. 1:**
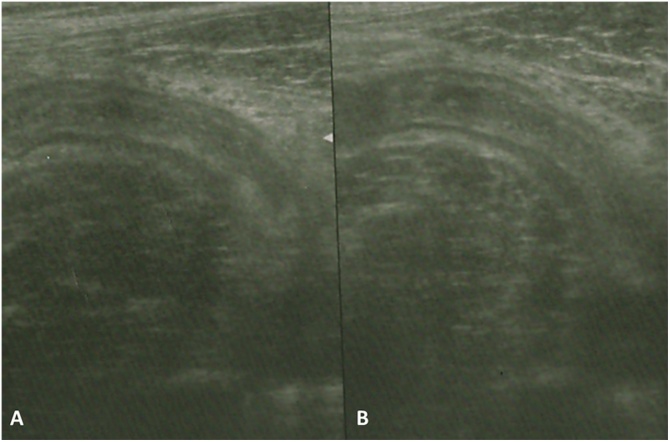
US scans (A and B) showing a tubular structure in the right iliac fossa, with thickened wall and a central area of inhomogeneous echogenicity.

An abdomino-pelvic ultrasonography (US) was performed. US shown a tubular structure in the right iliac fossa, with thickened wall and a central area of inhomogeneous echogenicity. Perilesional fluid layer was also detected (Fig. 1).

The US findings suggested an appendicitis, therefore the patient underwent CT examination.

A 320-row CT scanner was used (Multidetector CT Aquillon, Toshiba Medical System, Tokyo, Japan; detector collimation 0.5 mm, increment 0.5, 120/87 kVp/mAs).

CT acquisition was performed from the diaphragmatic domes to the pubic symphysis, using a triphasic technique after the intravenous injection of 1.5 mL/kg of Iopamidol (Iomeron 400; Bracco, Milan; Italy) at 3.5 mL/sec through the ante-cubital vein using an automatic power injector.

In order to evaluate bowel loops, a gastrografin solution, prepared by diluting 50 mL to one liter with water, was orally administered 20 min before the CT scan.

All CT data were transferred to a workstation (HP XW 8600) equipped with dedicated software for image reconstructions (Vitrea FX 2.1, Vital Images, Minneapolis, Minnesota, US).

Abdomino-pelvic CT showed a normal retrocecal appendix.

**Fig. 2:**
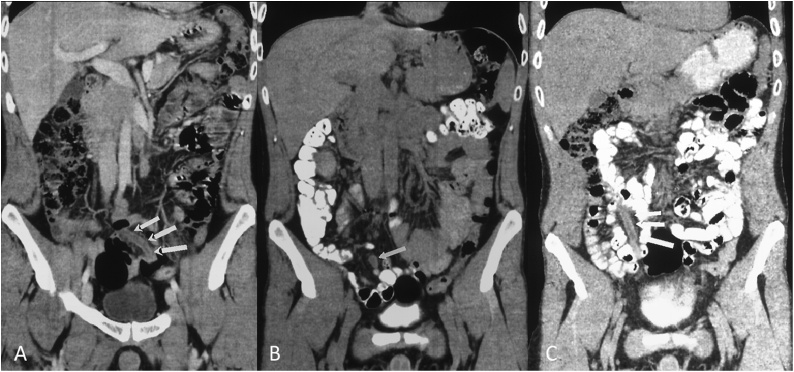
Coronal CT reconstructions after intravenous injection of contrast material (A) and hyperdense gastrointestinal contrast material administration (B and C) showing an hypodense tubular structure, 7 cm in length and 3 cm in width, strictly connected to a small bowel loop, with hyperdense thickened walls and perilesional fat strands into the adjacent mesentery (arrows).

The sonographic finding corresponded to a well-demarcated hypodense tubular structure, 7 cm in length and 3 cm in width, strictly connected to a small bowel loop. After the intravenous injection of contrast medium, the lesion demonstrated hyperdense thickened walls. Perilesional fat strands were also detected into the adjacent mesentery. No other pathologic findings were identified in the abdomino-pelvic district (Fig. 2).

The hypothesis of an inflamed Meckel’s diverticulum was performed, according to the tubular structure of the lesion, with hyperdense walls and perilesional mesenteric fat strands.

**Fig. 3:**
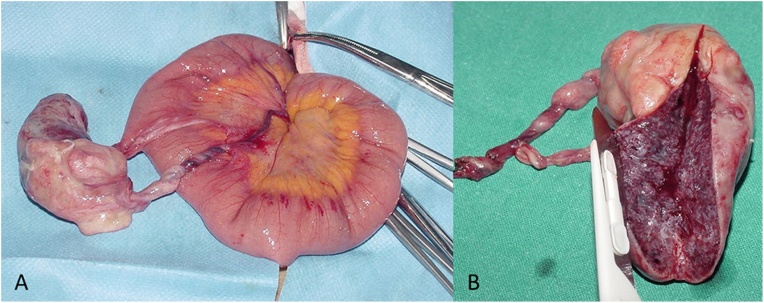
Surgical finding (A and B) after a laparotomic approach showing a mobile lesion connected to the small bowel mesentery by a fibrovascular pedicle. The lesion appeared as a fibrolipidic mass, showing vascular congestion due to the torsion around its pedicle causing the clinical onset.

The patient underwent surgery. After a laparotomic approach, the lesion was mobile in the peritoneal cavity, connected to the small bowel mesentery by a fibrovascular pedicle. The lesion appeared as a fibrolipidic mass, showing vascular congestion due to the torsion around its pedicle causing the clinical onset. Therefore, the pedicle was clamped and the mass was excised (Fig. 3).

Post-operatory course was uneventful and after a progressive improvement of the clinical condition, the patient was discharged after six days.

Histological examination revealed fat necrosis with lipid-filled macrophages associated to a dense lymphocyte infiltration and fibroblastic proliferation.

The CD117, SM-Actin, CD34, S-100 Protein markers were normally expressed, hence the pathological diagnosis was mesenteric lipodystrophy.

## 3 Discussion

Mesenteric lipodystrophy is a rare fibroinflammatory condition of the mesenteric fat of the abdomen. Since its first description by the Italian surgeon Jura in 1924, variations of the same pathological process have been reported under many names: sclerosing mesenteritis, mesenteric panniculitis, retractile mesenteritis, mesenteric lipodystrophy, mesenteric lipogranuloma1.

This ambiguous nomenclature reflects the progression from fat necrosis to fibrosis of the mesenteric adipose tissue.

In 1997, after a review of 84 cases, Emory et al. concluded that these histological variants are parts of the same disease and that “sclerosing mesenteritis” would have been an appropriate common nomenclature [1]. Nowadays most authors accept that these different entities represent a spectrum of the same disease.

Mesenteric involvement can be divided into three different types: diffuse thickening of the mesentery, knotty thickening of the root of mesentery and mass-like lesion.

Histologically, the mesenteric fat involved in this fibroinflammatory condition shows a progressive series of changes into three sequential phases. In the early phase of the process, the mesentery is characterized by fat necrosis with lipid-filled macrophage infiltration within the septa of the mesentery with absent or minimal inflammation, hence the name of mesenteric lipodystrophy [2].

Then, as the inflammation process progresses, fat necrosis is associated to a dense inflammatory cell infiltration (lymphocytes, plasma cell, histiocytes and giant cells). This aspect suggests the name of mesenteric panniculitis.

Finally, fibrosis occurs with consequent shortening and distortion of the mesentery. This final phase can be considered as an end stage process, a condition also known as retractile mesenteritis [3].

Mesenteric lipodystrophy affects most commonly patients from the fifth to seventh decades of life, with a male-female ratio of 2:1.

The incidence based on autopsy and CT findings varies from 0.4 to 1% [5].

The etiology is unknown. However, several conditions are suggested as potential causes: prior abdominal trauma or surgery, mesenteric ischemia, autoimmune diseases, tuberculosis, pancreatitis, vasculitis, granulomatous diseases and malignancies (colorectal cancer, lymphoma, cancers of urogenital tract) [6].

The correlation between mesenteric panniculitis and a neoplastic condition represents a controversial topic in the medical literature. In fact, many authors report that a correlation is possible by interpreting the involvement of the mesentery as a paraneoplastic syndrome. Scheer et al. [7] described a risk of malignancy 5 times higher in the presence of mesenteric panniculitis than in an inconspicuous mesentery; Van Puttie et al. [8] reported in their study that mesenteric panniculitis was associated with a significant higher prevalence of coexisting malignancies and patients with mesenteric panniculitis developed significantly more malignancies during a 5-year follow-up as compared with the control group.

Other authors do not recognize this correlation. About that, Gogebank et al., in a case–control study, refused the prevalent notion that mesenteric panniculitis is a paraneoplastic phenomenon and demonstrated that the degree and course of mesenteric panniculitis are not correlated with tumor stage or development [9]. Similarly, in a cohort study Buchwald et al. found no statistically significant association between cancer and mesenteric panniculitis, concluding that the latter is not a real paraneoplastic phenomenon but an epiphenomenon: CT and magnetic resonance imaging (MRI) are required mainly in neoplastic patients for staging and this would explain the high incidence of mesenteritis in cancer patients [10].

Sclerosing mesenteritis is sometimes associated with a multisystem clinical syndrome affecting the lungs, liver, biliary tract, pancreas and kidneys; this condition has been described as “IgG4-related systemic sclerosing disease” due to the presence of IgG4+ plasma cells within involved tissues [11].

The clinical presentation of sclerosing mesenteritis is heterogeneous and often non-specific, sometimes is totally asymptomatic and incidentally detected. Abdominal tenderness, nausea, vomiting, fever, fatigue are prevalent in the case of a diffuse mesenteric involvement; palpable abdominal mass and symptoms of bowel obstruction are described in the case of a mass forming process. If the mass is pedunculated, the torsion around the pedicle could be a fearing complication, compromising its vascularization and leading to ischemia [12,13].

Mesenteric lipodystrophy is often an incidental diagnosis; it shows 0.6% prevalence in patients undergoing abdominal computed tomography [4], which is the gold standard for the diagnosis of this condition.

Multiphasic CT with contrast enhanced technique is required in order to better characterize the lesion and to evaluate the degree of distortion of mesenteric vessels, which represent crucial informations for the surgical approach and for evaluating ischemic complications.

In order to assess the dislocation and compression of bowel loops, an oral administration of hydrosoluble contrast medium 20 min before the scan could be useful.

CT findings vary widely basing on the phase of fibroinflammatory process at the moment of diagnosis and on the main pathological aspects of mesenteric involvement.

A diffuse thickening of the mesentery is associated to increased fat density, which can be diffuse (“misty mesentery”) or can envelope the mesenteric vessels preserving a normal fatty area around them (“fat ring sign”). Mesenteric increased fat density is not specific for mesenteric lipodystrophy and can mimic other condition such as edema, carcinomatous lymphangitis and inflammatory bowel diseases [14].

A knotty thickening of the mesentery is associated to well-defined soft tissue nodules, usually less than 1 cm, associated to fat strand densities around the vessels. This condition must be differentiated from lymphadenopathies, tuberculosis, peritoneal carcinomatosis, mesothelioma and granulomatous diseases (Weber-Christian disease) [3].

A mass-like lesion often involve the small bowel mesentery. It is characterized by a fibrolipidic mass, usually surrounded by an hyperdense pseudocapsule (60% of cases), which may also have a sovrafluid component as a result of intralesional fat necrosis. Usually, in the central necrotic portion of the mass, calcification may be present and may be related to the fat necrosis [15].

These pseudotumors can grow into the mesentery or can be pedunculated; the latter are characterized by a fibrovascular pedicle and an esophitic growth into the peritoneal cavity. Torsion of the pedicle may cause severe ischemic complications [16].

Depending from their size and location, these abdominal masses are associated to various grade of mesenteric vessel distortion and various grade of dislocation or compression of the bowel loops [17].

Mesenteric lipodystrophy presenting as a mass lesion must be differentiated from a gastrointestinal stromal tumor (GIST), lymphoma, lipoma, liposarcoma, Meckel’s diverticulum.

Meckel's diverticulum is an intestinal diverticulum that results from incomplete obliteration of the most proximal portion of the vitelline or omphalo-mesenteric duct during the fifth week of fetal development [18]. The nonspecific CT findings need to be correlated to the clinical information and often requires biopsy and histological examination for a definitive diagnosis and a correct differential diagnosis with other pathological contidions [[19], [20], [21]].

Sonography isn’t useful for the study of mesentery; it can play a role only in detecting mesenteric mass, which are well defined and hyperechoic due to the fat content. The fatty content of the mass could reduce sonographic transmission, limiting the acquisition of information. If areas of necrosis occur, the mass shows central hypoecogenicity.

Ultrasonography is less invasive and money saving, but the lacking of panoramicity and the poor diagnostic information strongly limit its role into a first level examination.

As regards the therapy, there are not specific algorithms for mesenteric lipodystrophy; nowadays the treatment options are empiric and include the use of corticosteroids, colchicine, azathioprine, thalidomide, cyclophosphamide, tamoxifen and progesterone [11].

Surgical approach is reserved for complications such as bowel obstruction and vascular distortion with consequent ischemia of the mesenteric mass or ischemia of bowel loops [17,19].

## 4 Conclusions

Contrast enhanced CT is the most accurate imaging technique for diagnosing mesenteric lipodystrophy due to the high panoramicity and accuracy with multiplanar imaging. Multiphasic technique helps to characterize the lesion and to recognize vascular anatomy. Oral administration of iodinated contrast medium may help to assess the relationship with bowel loops.

All these diagnostic elements are crucial for the surgical timing and approach.

Due to the heterogeneous mesenteric involvement, the nonspecific CT findings and the high number of diseases for differential diagnosis, the detection of mesenteric lipodystrophy is challenging and requires early clinical suspicion. An histological examination is always necessary.

## Funding

None.

## Ethical approval

The study is exempt from ethical approval at our institution.

## Consent

Written informed consent obtained for case report and image publication.

## Author contribution

All authors conceived and designed the study, acquired the data, analysed and interpreted the data and drafted the manuscript. All authors revised the manuscript. All authors read and approved the final manuscript.

## Registration of research studies

This is not a first-in-man study.

## Guarantor

Alessandra Mirabile Marco Moschetta.

## Provenance and peer review

Not commissioned, externally peer-reviewed.

## Declaration of Competing Interest

None.

## References

[1] Emory T.S., Monihan J.M., Carr N.J., Sobin L.H. (1997). Sclerosing mesenteritis, mesenteric panniculitis and mesenteric lipodystrophy: a single entity?. Am. J. Surg. Pathol..

[2] McDermott R.L., Hutchinson B., Ryan C., Conneely J.B., Latif A., Maguire D. (2013). Mesenteric lipodystrophy - an unusual intraabdominal mass. Int. J. Surg. Case Rep..

[3] Vettoretto N., Diana D.R., Poiatti R., Matteucci A., Chioda C., Giovanetti M. (2007). Occasional finding of mesenteric lipodystrophy during laparoscopy: a difficult diagnosis. World J. Gastroenterol..

[4] Agha R.A., Borrelli M.R., Farwana R., Koshy K., Fowler A., Orgill D.P., For the SCARE Group (2018). The SCARE 2018 statement: updating consensus surgical CAse REport (SCARE) guidelines. Int. J. Surg..

[5] Daskalogiannaki M., Voloudaki A., Prassopoulos P., Magkanas E., Stefanaki K., Apostolaki E. (2000). CT evaluation of mesenteric panniculitis: prevalence and associated diseases. AJR Am. J. Roentgenol..

[6] Hussein M.R., Abdelwahed S.R. (2015). Mesenteric panniculitis: an update. Expert Rev. Gastroenterol. Hepatol..

[7] Scheer F., Spunar P., Wiggermann P., Wissgott C., Andresen R. (2016). Mesenteric Panniculitis (MP) in CT – a predictor of malignancy?. Fortschr Röntgenstr.

[8] Van Putte-Katie N., Van Bommel E.F.H., Elgersma O.E., Hendriksz T.R. (2014). Mesenteric panniculitis: prevalence, clinicoradiological presentation and 5-year follow-up. Br. J. Radiol..

[9] Gögebakan, Albrecht T., Osterhoff M.A., Reimann A. (2013). Is mesenteric panniculitis truely a paraneoplastic phenomenon? A matched pair analysis. Eur. J. Radiol..

[10] Buchwald P., Diesing L., Dixon L., Wakeman C., Eglinton T., Dobbs B. (2016). Frizelle cohort study of mesenteric panniculitis and its relationship to malignancy. Br. J. Surg..

[11] Bateman A.C., Deheragoda M.G. (2009). IgG4-related systemic sclerosing disease - an emerging and under-diagnosed condition. Histopathology.

[12] Edwards M.A., Smith M., Parker W., Gu K. (2007). Mesenteric lipodystrophy of the left colon. Am. Surg..

[13] Masulovic D., Jovanovic M., Ivanovic A., Stojakov D., Micev M., Stevic R. (2016). Sclerosing mesenteritis presenting as a pseudotumor of the greater omentum. Med. Princ. Pract..

[14] Avelino-Silva V.I., Leal F.E., Coelho-Netto C., Cotti G.C., Souza R.A., Azambuja R.L. (2012). Sclerosing mesenteritis as an unusual cause of fever of unknown origin: a case report and review. Clinics (Sao Paulo).

[15] Horton K.M., Lawler L.P., Fishman E.K. (2003). CT findings in sclerosing mesenteritis (Panniculitis): spectrum of disease. Radiographics.

[16] Rees J.R., Burgess P. (2010). Benign mesenteric lipodystrophy presenting as low abdominal pain: a case report. J. Med. Case Rep..

[17] Allen P.B., De Cruz P., Efthymiou M., Fox A., Taylor A.C., Desmond P.V. (2009). An interesting case of recurrent small bowel obstruction. Case Rep. Gastroenterol..

[18] Kassir R., Debs T., Boutet C., Baccot S., Abboud K., Dubois J. (2015). Intussusception of the Meckel’s diverticulum within its own lumen: unknown complication. Int. J. Surg. Case Rep..

[19] Angelelli G., Moschetta M., Cosmo T., Binetti F., Scardapane A., Stabile Ianora A.A. (2012). CT diagnosis of the nature of bowel obstruction: morphological evaluation of the transition point. Radiol. Med..

[20] Johnson P.T., Horton K.M., Fishman E.K. (2009). Nonvascular mesenteric disease: utility of multidetector CT with 3D volume rendering. Radiographics.

[21] Angelelli G., Moschetta M., Sabato L., Morella M., Scardapane A., Stabile Ianora A.A. (2011). Value of “protruding lips” sign in malignant bowel obstructions. Eur. J. Radiol..

